# Preparation and Mechanistic Characterization of α-Glucosidase Inhibitory Peptides from *Elaeagnus mollis* Oilseed Meal

**DOI:** 10.3390/foods15081323

**Published:** 2026-04-10

**Authors:** Caixia Guo, Tong Wen, Xuefeng Tian, Meiping Li, Ligang Yu, Tingting Zhang

**Affiliations:** 1 School of Life Science, Shanxi University, Taiyuan 030006, China; guocx@sxu.edu.cn (C.G.); wentong@sxu.edu.cn (T.W.); t18536225545@163.com (X.T.); lmpmg@sxu.edu.cn (M.L.); yuligang@sxu.edu.cn (L.Y.); 2 Research Institute of Applied Biology, Shanxi University, South Zhonghuan East Street No. 63, Taiyuan 030006, China

**Keywords:** *Elaeagnus mollis* oilseed meal, peptides, α-glucosidase inhibitor, ultrafiltration, stability, structural characterization, inhibitory mechanism, cytotoxicity assessment

## Abstract

*Elaeagnus mollis* oilseed (EMO) meal is a protein-rich by-product that may serve as a novel source of food-derived α-glucosidase inhibitory peptides. This study aimed to obtain EMO peptide fractions with enhanced α-glucosidase inhibition and to clarify the activity, stability and mechanism of the most active fraction. Fourteen proteases were compared, and 3.350 acidic protease was selected to establish an optimized hydrolysis process. The resulting EMO hydrolysate showed an IC_50_ of 9.11 mg/mL against α-glucosidase and no detectable cytotoxicity towards HEK-293T cells at 0.1–12.0 mg/mL. Ultrafiltration yielded four fractions, among which the 3–10 kDa fraction exhibited the highest inhibition and maintained substantial activity under acidic pH (2–6), −20–50 °C, NaCl ≤ 5% and simulated gastrointestinal digestion. Kinetic analysis indicated mixed-type inhibition, while fluorescence, circular dichroism and molecular docking suggested that peptides in this fraction bind near the catalytic site of α-glucosidase and induce local conformational changes. These findings support EMO-derived 3–10 kDa peptides as stable, non-cytotoxic α-glucosidase inhibitors with potential as functional ingredients for dietary management of type 2 diabetes.

## 1. Introduction

Diabetes mellitus is a group of metabolic disorders characterized by chronic hyperglycemia resulting from impaired insulin secretion or impaired insulin action. In 2021, an estimated 537 million adults worldwide were living with diabetes, and this number is projected to reach 853 million by 2050 [1], making it a major global public health and socioeconomic challenge. Clinically, diabetes is classified into type 1, type 2, gestational diabetes and other specific types according to pancreatic islet function and underlying aetiology, with type 2 diabetes accounting for approximately 90% of all cases [2]. Type 2 diabetes is a multifactorial disorder driven by genetic susceptibility, sedentary lifestyles, chronic psychosocial stress and excess energy intake [3]. Its management relies largely on a combination of dietary control, regular physical activity and oral hypoglycemic agents. Among these, α-glucosidase inhibitors act by targeting α-glucosidase located in the brush border of the small intestinal mucosa, thereby delaying the conversion of dietary polysaccharides and oligosaccharides into absorbable monosaccharides and effectively blunting the postprandial rise in blood glucose. Compared with drugs that directly stimulate insulin secretion, this class of inhibitors modulates carbohydrate digestion at the intestinal level and may help to reduce the functional burden on pancreatic β-cells [4]. However, currently used α-glucosidase inhibitors such as acarbose, miglitol and voglibose are frequently associated with gastrointestinal adverse effects, which limits long-term adherence [5]. A growing body of evidence has shown that many natural products and their extracts contain components with marked α-glucosidase inhibitory activity and minimal toxicity, highlighting natural matrices as promising sources for the development of new α-glucosidase inhibitors for type 2 diabetes management [6,7,8].

In recent years, peptides with α-glucosidase inhibitory activity have attracted increasing attention as candidates for dietary management of type 2 diabetes. Such peptides have been identified from a wide range of biological sources, including plant, animal and marine proteins, and are generally characterized by good safety and reasonable stability. For example, α-glucosidase inhibitory peptides have been reported from peanut meal protein, shiitake mushroom, earthworm protein, sweet potato protein, spirulina, ginkgo seed cake protein, buffalo milk proteins and Semen Ziziphi Spinosae protein [9,10,11,12,13,14,15,16], among others, supporting the feasibility of using food proteins as precursors of α-glucosidase inhibitory peptides. *Elaeagnus mollis* is a woody oilseed species with recognized nutritional and medicinal value; its kernels are rich in lipids, proteins, vitamins and minerals. During industrial oil production, pressing of the kernels generates *E. mollis* oilseed (EMO) meal, a pale, powdery by-product that still retains a high protein content and a substantial proportion of total amino acids, but is currently used mainly as low-value feed or discarded. Our previous work has shown that protein isolates prepared from EMO meal exhibit good solubility and interfacial properties, and that controlled enzymatic hydrolysis can reduce molecular weight, modulate protein conformation and enhance techno-functional and antioxidant properties [17]. These findings indicate that EMO meal protein is a promising substrate for the production of bioactive peptides. Nevertheless, α-glucosidase inhibitory peptides derived from this by-product have not yet been systematically explored. Mining and characterizing α-glucosidase inhibitory peptides from EMO meal would therefore not only provide new food-derived candidates for glycemic control, but also offer a route to the value-added utilization of an underused oilseed processing residue.

In this study, we established an enzymatic hydrolysis process for EMO meal protein to obtain peptide hydrolysates with enhanced α-glucosidase inhibitory activity. Ultrafiltration was then used to concentrate peptide fractions with higher inhibitory potency, which were further evaluated in terms of cytotoxicity and the stability of α-glucosidase inhibition under relevant pH, temperature, ionic strength and simulated gastrointestinal conditions. By combining enzyme kinetics and spectroscopic analyses with LC-MS/MS-based peptide identification and molecular docking, we examined the relationships between peptide composition, structure and inhibitory activity and clarified the inhibitory mode of the most active fraction. This work aims to identify safe and effective α-glucosidase inhibitory peptides from EMO meal and to provide a scientific basis for the value-added utilization of this by-product as a candidate ingredient for dietary support in the management of type 2 diabetes.

## 2. Materials and Methods

### 2.1. Materials and Reagents

EMO meal, a by-product obtained after supercritical carbon dioxide extraction of the oil, was supplied by Shanxi Qierkang Samara Biological Products Co., Ltd., Yuncheng, China. α-Glucosidase, p-nitrophenyl-α-D-glucopyranoside (pNPG) and dimethyl sulfoxide (DMSO) were purchased from Shanghai Yuanye Bio-Technology Co., Ltd., Shanghai, China. Acarbose, glutathione (GSH), 537 acidic protease (200,000 U/g and 20,000 U/g), Flavour protease (100,000 U/g), pepsin (3000 U/mg), bromelain (850,000 U/g) and high-temperature neutral protease 9530 (11,000 U/g) were obtained from Guangzhou Tanjia Bio-Technology Co., Ltd., Guangzhou, China. 2,2-Diphenyl-1-picrylhydrazyl (DPPH), 2,2′-azino-bis(3-ethylbenzothiazoline-6-sulfonic acid) diammonium salt (ABTS), Solarbio neutral protease (50,000 U/g), Solarbio acidic protease (50,000 U/g), bovine hemoglobin, vitamin B12, bacitracin zinc, myoglobin and aprotinin were purchased from Beijing Solarbio Science & Technology Co., Ltd., Beijing, China. 3.350 acidic protease (43,000 U/g) was purchased from Tianjin Nuoao Technology Development Co., Ltd., Tianjin, China; Longda acidic protease (800,000 U/g, 700,000 U/g, 35,000 U/g) from Shandong Longda Bioengineering Co., Ltd., Yishui, China; and Amano neutral protease (55,000 U/g) from Hebei Tuohai Biotechnology Co., Ltd., Shijiazhuang, China. Sephadex G-50 was purchased from Shanghai Lanji Technology Development Co., Ltd., Shanghai, China. All other chemicals were of analytical grade and obtained from local chemical suppliers.

### 2.2. Preparation of EMO Peptides

EMO meal (20 g) was dispersed in 200 mL distilled water (solid-to-liquid ratio of 1:10, *w*/*v*), and sodium bisulfite (NaHSO_3_, 0.35% *w*/*w*, based on EMO meal) was added. The pH of the suspension was adjusted to 4.0 using an equal-volume mixture of 5% sulfuric acid and 5% phosphoric acid, and the mixture was soaked overnight at room temperature. After soaking, the slurry was homogenized at high speed for 2 min. The homogenizer was rinsed with 50 mL of distilled water, and the rinse was combined with the homogenate to obtain a final solid-to-liquid ratio of 1:12.5 (*w*/*v*). The mixture was then heated to 85 °C for 3 min to facilitate protein release, cooled to room temperature and used immediately for enzymatic hydrolysis.

In this study, EMO meal was hydrolyzed using 14 commercially available and laboratory accessible acidic and neutral proteases. Enzymatic hydrolysis was performed under the optimal conditions recommended for each protease (Table 1), and these conditions were set according to the manufacturers’ recommendations or our laboratory experience. Briefly, 54 g of the pretreated EMO slurry was adjusted to the optimal pH of each protease with the mixed acid solution and 1.0 mol/L NaOH. Hydrolysis was initiated by adding protease at 5000 U/g and maintained at the corresponding optimal temperature in a water bath for 1 h. The enzymatic reaction was terminated by boiling for 10 min. After cooling to room temperature, the mixture was centrifuged at 4000 rpm for 20 min, and the supernatant was collected as the EMO peptides solution. Peptide yield and α-glucosidase inhibitory activity of the EMO peptides were determined to identify proteases with high hydrolysis efficiency, which were then subjected to further optimization.

The effects of substrate concentration, enzyme dosage, pH, hydrolysis temperature and hydrolysis time on the α-glucosidase inhibitory activity and peptide yield of EMO peptides were first evaluated in single-factor experiments. On this basis, an orthogonal experimental design was applied to systematically optimize the enzymatic hydrolysis conditions. Four key factors, namely substrate concentration, enzyme dosage, pH and hydrolysis time, were each set at three levels, and an L9(3^4^) orthogonal array was used to assess their combined effects on α-glucosidase inhibitory activity and peptide yield.

### 2.3. Determination of Peptide Yield

Peptide yield was determined according to a previously established method with minor modifications [18]. Briefly, 2 mL of peptide solution was mixed with an equal volume of 20% (*w*/*v*) trichloroacetic acid, kept at room temperature for 10 min and centrifuged at 5000 rpm for 10 min. An aliquot of the supernatant (1 mL) was then mixed with 4 mL of biuret reagent, incubated at room temperature for 30 min, and the absorbance was measured at 540 nm using distilled water as the blank. A calibration curve was constructed using bovine serum albumin (BSA) as the standard. The concentration range of the BSA standard solutions was 0.0–10.0 mg/mL, and the regression equation relating absorbance (y) to BSA concentration (x, mg/mL) was y = 0.045x + 0.0008 (R^2^ = 0.9999). The soluble protein content of the peptide solution was calculated from this standard curve. In addition, the free amino nitrogen content was determined by the potentiometric method according to the Chinese National Standard GB 5009.235-2016 [19]. Peptide yield was calculated using the following equation:

$$Peptide\;yield\;\left(\%\right)=\frac{C_{1}-C_{2}\times 5.83}{C_{0}}\times 100$$

where C_0_, C_1_ and C_2_ are the total protein, soluble protein and free amino nitrogen contents of the samples, respectively (all expressed in mg/mL), and 5.83 was used as the nitrogen to protein conversion factor to convert free amino nitrogen into protein equivalent content, thereby allowing the correction term to be expressed on the same basis as soluble protein content in the calculation of peptide yield.

### 2.4. Determination of α-Glucosidase Inhibitory Activity

The α-glucosidase inhibitory activity was determined according to the method of Rivero et al. [20], with minor modifications. Briefly, 0.2 mL of sample solution was mixed with 0.2 mL of α-glucosidase solution (0.2 U/mL). Phosphate buffer (0.1 mol/L, pH 6.8) was added to adjust the reaction volume to 0.9 mL, and the mixture was incubated at 37 °C for 10 min. Then 0.1 mL of pNPG solution (3.0 mmol/L) was added and the reaction was allowed to proceed at 37 °C for 20 min. The reaction was stopped by adding 1.5 mL of Na_2_CO_3_ solution (0.1 mol/L), and the absorbance was measured at 405 nm. Acarbose was used as the positive control at concentrations of 0.001, 0.005, 0.010, 0.015, 0.020, and 0.025 mg/mL, and was assayed under the same conditions. The inhibition rate was calculated as:

$$\alpha -glucosidase\;inhibition\;\left(\%\right)=(1-\frac{A_{4}-A_{3}}{A_{2}-A_{1}})\times 100$$

where A_1_ is the absorbance of the blank, A_2_ is the absorbance of the control (enzyme and substrate without sample), A_3_ is the absorbance of the sample blank, and A_4_ is the absorbance of the sample reaction mixture.

### 2.5. Cytotoxicity of EMO Peptides

The cytotoxicity of EMO peptides was evaluated in HEK-293T cells using the MTT assay as previously established method with minor modifications [21], with minor modifications. EMO peptide solutions were prepared at concentrations ranging from 0.1 to 12.0 mg/mL, and the maximum concentration was selected based on the α-glucosidase inhibitory activity of EMO peptides to cover and slightly exceed the IC_50_-related functional concentration range. HEK-293T cells were seeded in 96-well plates at 1 × 10^5^ cells/mL and incubated for 24 h to allow attachment. The culture medium was then replaced with medium containing different concentrations of EMO peptides, and cells were further incubated. Subsequently, MTT reagent and fresh culture medium were added and cells were incubated for 4 h, after which the supernatant was removed and DMSO was added to dissolve the formazan crystals. The plates were shaken for 10 min and the absorbance was measured at 490 nm. Acarbose was used as a positive control. Cell viability was calculated based on the absorbance of treated wells relative to the untreated control.

### 2.6. Determination of the Molecular Weight Distribution of EMO Peptides

The molecular weight distribution of α-glucosidase inhibitory peptides from EMO was determined by gel filtration chromatography on Sephadex G-50 according to Guo et al. [18], with minor modifications. Sephadex G-50 (25 g) was allowed to swell in distilled water for 24 h, washed repeatedly to remove impurities and floating particles, boiled in a water bath to eliminate air bubbles and then cooled for use. The swollen gel was packed into a column under gravity to form a stable bed with a height of approximately 10 cm below the column top, and the column was equilibrated with Tris–HCl buffer (pH 7.2) at a flow rate 1.33 times that used for sample elution. Before sample loading, the column outlet was opened until the liquid level reached the top of the gel bed and then closed. Crude EMO peptides, prefiltered through a 0.22 μm membrane, was loaded onto the column (1 mL) along the wall. After the sample entered the gel bed, about 5 mL of Tris–HCl buffer (pH 7.2) was added to the column top and elution was carried out at a flow rate of 0.6 mL/min using a peristaltic pump. Elution was monitored at 220 nm, and the relative proportions of peptides in different molecular weight regions were calculated by peak-area normalization.

### 2.7. Ultrafiltration of EMO Peptides

The EMO peptides was transferred into ultrafiltration centrifugal tubes with molecular weight cut-offs of 10 kDa, 3 kDa and 1 kDa and centrifuged at 4 °C and 4000 rpm for fractionation. Four peptide fractions were collected according to the membrane cut-offs, corresponding to >10 kDa, 3–10 kDa, 1–3 kDa and <1 kDa, and were stored at 4 °C until use.

### 2.8. Characterization of EMO Peptide Fractions

#### 2.8.1. Surface Hydrophobicity

Surface hydrophobicity of the different EMO peptide fractions was determined using ANS fluorescence according to Li et al. [22], with minor modifications. Each fraction was dissolved to obtain peptide solutions at 0.05, 0.10, 0.15, 0.20 and 0.25 mg/mL. An aliquot of 4 mL of peptide solution was mixed with 20 μL of ANS solution (8 mmol/L) and incubated at room temperature in the dark for 15 min. Fluorescence intensity was measured at an excitation wavelength of 390 nm and an emission wavelength of 530 nm with a slit width of 5 nm. A scatter plot of fluorescence intensity versus peptide concentration was generated for each fraction, and the slope of the linear regression was taken as the surface hydrophobicity index.

#### 2.8.2. Intrinsic Fluorescence Spectroscopy

Intrinsic fluorescence spectra of the EMO peptide fractions were recorded as described by Xiong et al. [23], with slight modifications. Each fraction was dissolved to a final concentration of 0.01 mg/mL. Samples were excited at 280 nm with a slit width of 5 nm, and emission spectra were scanned from 300 to 500 nm.

#### 2.8.3. UV Absorption Spectroscopy

UV absorption spectra of the different EMO peptide fractions were measured following the method of Xiong et al. [23], with minor modifications. Peptide solutions of each fraction were prepared at 1.0 mg/mL, and UV spectra were recorded in the range of 200–400 nm.

#### 2.8.4. Infrared Spectroscopy

Infrared spectra of the EMO peptide fractions were obtained according to Du et al. [24]. Lyophilized powders of each fraction were used directly for analysis with an FTIR spectrometer. Spectra were collected over a wavenumber range of 7800–350 cm^−1^ with a spectral resolution better than 0.09 cm^−1^ to characterize the functional groups of the peptides.

### 2.9. Stability of EMO Peptides

#### 2.9.1. Effect of Temperature on Inhibitory Activity

The thermal stability of EMO peptide fractions was evaluated according to Li et al. [25], with minor modifications. EMO peptide fractions were adjusted to 10 mg/mL and incubated at −20, 4, 25, 37, 50, 70 and 90 °C for 30 min. Samples were then brought back to room temperature, and the α-glucosidase inhibition of each fraction was determined as described above.

#### 2.9.2. Effect of pH on Inhibitory Activity

The effect of pH on the inhibitory activity of EMO peptide fractions was evaluated according to Li et al. [25], with minor modifications. Each peptide fraction was prepared at 10 mg/mL, and the pH of each fraction was adjusted to 2, 3, 4, 6, 8 or 10 using HCl or NaOH. After incubation at room temperature for 1 h, the α-glucosidase inhibitory activity of each fraction was measured to evaluate pH stability.

#### 2.9.3. Effect of NaCl on Inhibitory Activity

The effect of ionic strength on the inhibitory activity of EMO peptide fractions was evaluated according to Liu et al. [26], with minor modifications. Peptide fractions were prepared at 15 mg/mL. An aliquot of 1.0 mL of peptide solution was mixed with 0.5 mL of NaCl solution at different mass fractions (0.5%, 1.0%, 2.0%, 3.0%, 4.0% and 5.0%). After mixing, the samples were kept at room temperature for 1 h, α-glucosidase inhibitory activity was determined to assess the influence of ionic strength.

#### 2.9.4. In Vitro Digestion

The in vitro digestive stability of EMO peptide fractions was evaluated according to Wu et al. [27], with minor modifications. Each fraction was adjusted to 10 mg/mL, and the pH was set to 2.0 with 1.0 mol/L HCl. Samples were pre-incubated in a 37 °C water bath for 20 min to simulate gastric conditions, followed by the addition of pepsin at 2.5% (*w*/*w*, based on substrate). After incubation at 37 °C for 2 h, the reaction was stopped by adjusting the pH to 7.5 with 1.0 mol/L NaOH. An aliquot of 1.5 mL of the pepsin digest was then taken for the determination of α-glucosidase inhibitory activity. Trypsin was then added to the digested solution at 4% (*w*/*w*, based on substrate) and the mixture was incubated at 37 °C for a further 2 h. The reaction was terminated by heating in a boiling water bath for 10 min, and α-glucosidase inhibitory activity of the trypsin digests was measured to assess the stability of the peptide fractions under simulated gastric and intestinal digestion.

### 2.10. Peptide Identification

According to the method of Feng et al. [28], the amino acid sequences of the 3–10 kDa fraction of EMO α-glucosidase inhibitory peptides were identified by LC-MS/MS. Before LC–MS/MS analysis, the peptide sample was desalted using Empore^TM^ SPE Cartridges C18 (standard density, CDS Analytical LLC, Oxford, PA, USA) and lyophilized. The lyophilized sample was then dissolved in sample solvent containing 0.1% formic acid and 2% acetonitrile, centrifuged at 17,000× *g* at 4 °C for 15 min, and the supernatant was collected for LC-MS/MS analysis. Samples were separated on an EASY-nLC 1000 system (Thermo Fisher Scientific Inc., Waltham, MA, USA) equipped with an Acclaim PepMap C18 trap column (100 μm × 2 cm) and an Acclaim PepMap C18 analytical column (75 μm × 250 mm) (Thermo Fisher Scientific Inc., Waltham, MA, USA) with 0.1% (*v*/*v*) formic acid in water (solvent A) and 0.1% (*v*/*v*) formic acid in 80% (*v*/*v*) acetonitrile (solvent B) at a flow rate of 300 nL/min. Mass spectrometric data were acquired on a Thermo Orbitrap Fusion mass spectrometer (Thermo Scientific, Thermo Fisher Scientific Inc., Waltham, MA, USA) in positive ion mode. The spray voltage was set at 2.2 kV, and the cycle time was 1.3 s. Full MS scans were acquired over an *m*/*z* range of 350–1500 at a resolution of 120 K, with an AGC target of 1 × 10^5^ and a maximum injection time of 50 ms. MS/MS spectra were acquired in the ion trap with an isolation window of 1.6 Th, an AGC target of 1 × 10^4^, a maximum injection time of 35 ms, and HCD activation at a collision energy of 35. Raw data were processed using PEAKS 8.5 for de novo sequencing and database searching against the homologous database Elaeagnaceae.fasta downloaded from NCBI to obtain the amino acid sequences of EMO peptides.

### 2.11. Mechanism of α-Glucosidase Inhibition by EMO Peptides

#### 2.11.1. Enzyme Kinetics of α-Glucosidase Inhibition

The inhibition kinetics of α-glucosidase were investigated according to Chen et al. [29], with minor modifications. First, the substrate pNPG concentration was fixed at 3.0 mmol/L and the enzyme concentration was varied (0.15, 0.25, 0.35, 0.45 and 0.55 U/mL) in the absence or presence of the 3–10 kDa peptide fraction at 0, 4.0, 8.0 and 12.0 mg/mL. Initial reaction rates (v) were determined from the change in absorbance at 405 nm, and v was plotted against enzyme concentration to assess the reversibility of inhibition. In a second set of experiments, the α-glucosidase concentration was fixed at 0.2 U/mL and reactions were carried out at different substrate concentrations (0.5, 1.0, 2.0, 3.0 and 4.0 mmol/L) with the same peptide concentrations (0, 4.0, 8.0 and 12.0 mg/mL). Lineweaver–Burk plots were constructed by plotting 1/[S] versus 1/v, and the inhibition type was determined from the pattern of the fitted lines.

#### 2.11.2. Fluorescence Quenching Between EMO Peptides and α-Glucosidase

Mixed solutions were prepared in phosphate buffer to a final volume of 3 mL, containing α-glucosidase at 0.05 mg/mL and peptides at 4.0, 4.5, 5.0, 6.0, 8.0 or 12.0 mg/mL. After mixing, samples were analyzed on a fluorescence spectrophotometer with an excitation wavelength of 280 nm and a slit width of 5 nm. Emission spectra were recorded from 300 to 500 nm, and changes in fluorescence intensity were used to evaluate peptide-induced quenching of the enzyme.

#### 2.11.3. Circular Dichroism Analysis of α-Glucosidase Secondary Structure

Peptide and enzyme mixtures were prepared in phosphate buffer at peptide-to-enzyme mass ratios of 0:1, 5:1, 10:1 and 15:1 and incubated for 10 min. Far-UV CD spectra were then recorded from 180 to 260 nm at a scan rate of 100 nm/min. Spectral changes were analyzed to assess alterations in the secondary structure of α-glucosidase upon peptide binding.

#### 2.11.4. Molecular Docking of EMO Peptides with α-Glucosidase

To characterize peptide–enzyme interactions at the molecular level, molecular docking was performed between the identified peptides and α-glucosidase, with acarbose used as a reference ligand. The crystal structure of *Saccharomyces cerevisiae* α-glucosidase (PDB ID: 3A4A) was obtained from the Protein Data Bank and used as the receptor. The receptor structure was pre-processed in PyMOL (version 3.1.0) by removing water molecules and adding hydrogen atoms. The 34 peptide sequences identified in this study were constructed as ligand structures in PyMOL and energy-minimized using PyRx 8.0. The structure of acarbose was obtained from the PubChem database (CID: 9811704) and prepared using the same docking procedure. The receptor and ligand structures were imported into AutoDock Vina (version 1.2.7), and the docking box was defined around the active site before docking calculations were performed. The resulting complexes were visualized in PyMOL to inspect three-dimensional binding poses, and two-dimensional interaction diagrams were generated using LIGPLOT (version 2.3) to identify key residues, hydrogen bonds, and hydrophobic contacts involved in the interaction between ligands and α-glucosidase.

### 2.12. Data Analysis

Data were analyzed using SPSS Statistics 26.0 (IBM Corp., Armonk, NY, USA) and are presented as mean ± standard deviation. Differences among groups were assessed by one-way ANOVA, and *p* < 0.05 was considered statistically significant. Figures were prepared using Origin 2022 (OriginLab Corp., Northampton, MA, USA).

## 3. Results and Discussion

### 3.1. Selection of Protease and Hydrolysis Conditions for EMO Meal

To obtain EMO meal-derived peptides with high α-glucosidase inhibitory activity, fourteen commercial proteases were initially compared under their respective optimal reaction conditions. Peptide yield and α-glucosidase inhibitory activity were used as evaluation indices. As summarized in Table 2, after 1 h of hydrolysis under the optimal conditions for each protease, peptide yields ranged from 18.48% to 58.68%, and α-glucosidase inhibitory activity ranged from 11.72% to 43.38%. Among all proteases, hydrolysates produced by 3.350 acidic protease showed the highest peptide yield (58.68%) and α-glucosidase inhibition (43.38%), whereas Amano neutral protease gave the lowest peptide yield (18.48%) and the *Bacillus* neutral protease showed the lowest α-glucosidase inhibition (11.72%). These results reflect that differences in catalytic activity and cleavage specificity among proteases determine the extent of protein breakdown as well as the molecular-weight distribution and amino acid composition of the resulting peptides, which in turn markedly affect their biological activities [30,31]. Similar trends have also been reported for insect proteins, edible mushroom proteins and seed or legume proteins, where changing the protease type or using different protease combinations markedly altered peptide size distributions and sequences, with corresponding changes in bioactivities [32,33,34,35,36]. Taken together, these findings indicate that rational selection of proteases is a critical step for obtaining peptides with strong antidiabetic potential. On this basis, 3.350 acidic protease was selected as the enzyme preparation for producing α-glucosidase inhibitory peptides from EMO meal.

**Figure 1:**
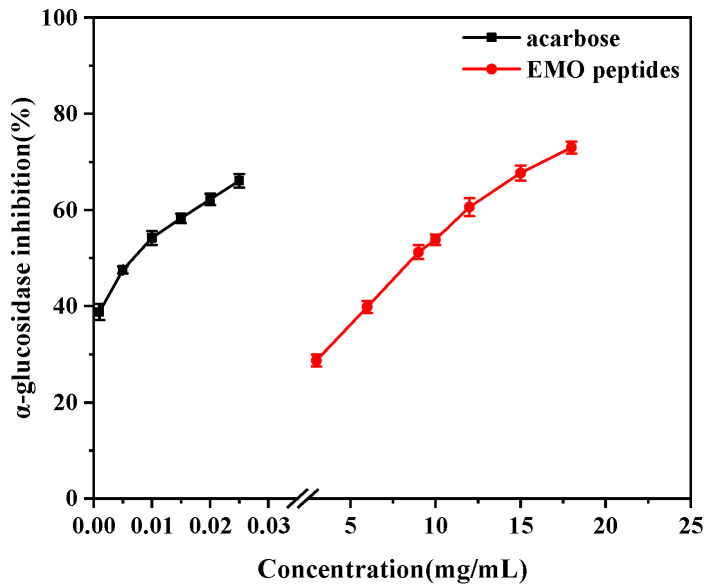
α-Glucosidase inhibitory activity of EMO peptides obtained under the optimal hydrolysis conditions at different peptide concentrations.

Subsequently, hydrolysis conditions for EMO meal with 3.350 acidic protease were further optimized. Single-factor experiments combined with an orthogonal design were employed, and the detailed factor settings and statistical analyses are provided in Supplementary Material S1. Considering both peptide yield and α-glucosidase inhibition, the optimal hydrolysis conditions were determined as follows: substrate concentration 4% (*w*/*v*), enzyme dosage 7000 U/g, pH 3.0, reaction time 3 h and hydrolysis temperature 50 °C. Under these conditions, the peptide yield reached 86.54% and the α-glucosidase inhibition rate was 56.30%. The α-glucosidase inhibitory activity of EMO peptides obtained under the optimal conditions was then evaluated at different peptide concentrations (Figure 1). The inhibitory effect increased with increasing peptide concentration, showing a clear concentration-dependent pattern, and the IC_50_ value was 9.11 mg/mL. This value was markedly higher than that of acarbose (IC_50_ = 0.0085 mg/mL), indicating a weaker inhibitory activity than the positive control. These optimized conditions were therefore used in all subsequent experiments to prepare EMO meal-derived α-glucosidase inhibitory peptides.

### 3.2. Evaluation of EMO Peptide Cytotoxicity in HEK-293T Cells

**Figure 2:**
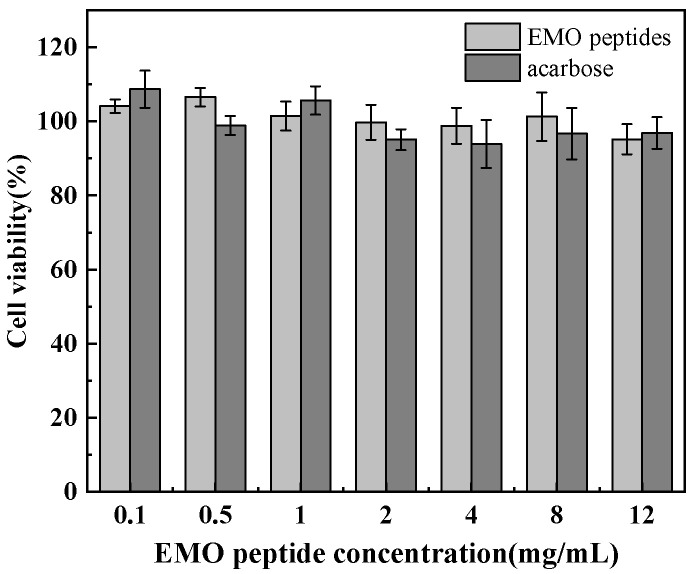
Cytotoxicity assessment of EMO peptides in HEK-293T cells.

To evaluate potential cytotoxicity, we examined the effects of EMO peptides on HEK-293T cells at concentrations ranging from 0.1 to 12.0 mg/mL. As shown in Figure 2, cell viability in the presence of EMO peptides remained within approximately 93–105% across this concentration range, while acarbose at the same concentrations resulted in viabilities of about 95–108%, with only slight fluctuations as concentration increased. Previous studies have suggested that, in cell-based cytotoxicity assays, test samples can generally be regarded as non-cytotoxic when cell viability remains above 90% [37]. Taken together, these results indicate that EMO peptides do not exhibit detectable cytotoxicity within the tested concentration range and support their further exploration as safe functional ingredients for the development of glucose-lowering foods within this concentration range under in vitro conditions. However, the present data do not define the upper toxic limit or a broader safety window, and their in vivo safety still requires further investigation.

### 3.3. Molecular Weight Distribution of EMO Peptides

**Figure 3:**
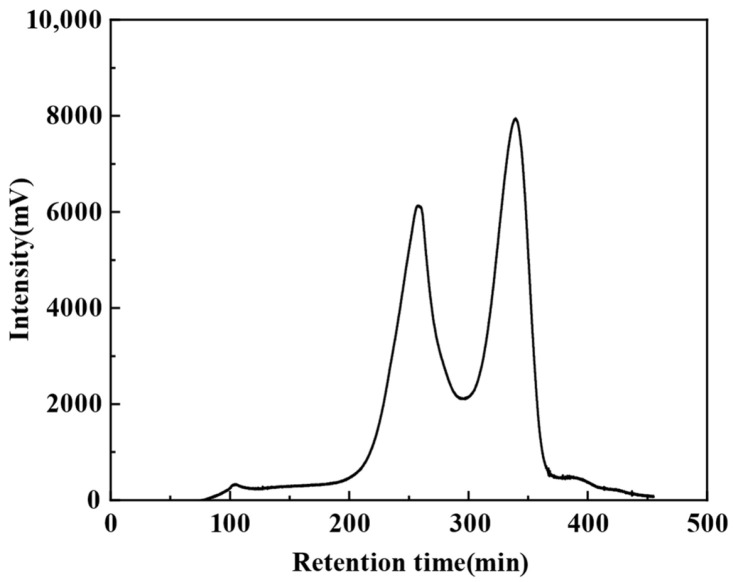
Sephadex G-50 gel filtration chromatogram of EMO peptides.

The molecular weight distribution of EMO peptides was analyzed using a Sephadex G-50 column. A standard curve was constructed with five standard proteins by plotting elution volume (Ve) against the logarithm of molecular weight (log Mr), giving a linear regression equation of y = −49.86x + 299.50 (R^2^ = 0.9906). The elution profile of EMO peptides on the Sephadex G-50 column is shown in Figure 3. Based on peak-area normalization and the standard curve, peptides with molecular weights >10 kDa accounted for 6.59% of the total. The 3–10 kDa and 1–3 kDa fractions represented 5.32% and 12.48%, respectively, while peptides <1 kDa accounted for 75.61%. Overall, these results indicate that the hydrolysate is mainly composed of small peptides (<10 kDa), especially those <1 kDa, demonstrating that 3.350 acidic protease efficiently converts EMO proteins into short-chain peptides.

### 3.4. Ultrafiltration Fractionation

**Figure 4:**
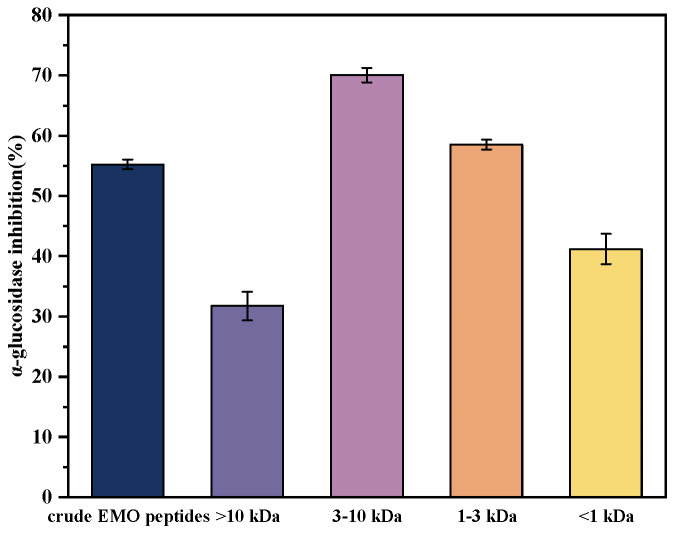
α-Glucosidase inhibitory activity of EMO peptide fractions with different molecular-weight ranges (>10 kDa, 3–10 kDa, 1–3 kDa, and <1 kDa).

Based on the previous molecular weight distribution, ultrafiltration was employed to further clarify the contribution of different molecular weight ranges to the α-glucosidase inhibitory effect of EMO peptides. The peptides were fractionated using membranes with nominal cut-off values of 10, 3 and 1 kDa to obtain four fractions (>10 kDa, 3–10 kDa, 1–3 kDa and <1 kDa). Each fraction was adjusted to 10 mg/mL, and its α-glucosidase inhibitory activity was determined (Figure 4). The results showed that the α-glucosidase inhibitory activity of the four fractions differed significantly (*p* < 0.05). The 3–10 kDa fraction exhibited the highest inhibition, reaching 70.03%, followed by the 1–3 kDa fraction, while the crude EMO peptides showed an intermediate level. In contrast, the <1 kDa fraction displayed a lower inhibitory activity than the crude peptides. These results indicate that excessive hydrolysis, where peptides are further cleaved into smaller fragments (<1 kDa), can lead to additional degradation of functional peptides and, consequently, a reduction in α-glucosidase inhibitory activity, in line with previous observations in other protein hydrolysate systems [36]. Overall, the main contribution to the α-glucosidase inhibitory effect of EMO peptides came from the medium molecular weight fractions, especially the 3–10 kDa fraction. Similar results have been reported for other proteins, where peptides derived from Ginkgo biloba seed cake in the <3 kDa and walnut protein hydrolysates in the 3–10 kDa range showed stronger α-glucosidase inhibitory activity [14,38]. These studies support medium molecular weight fractions such as 3–10 kDa as an important source of effective α-glucosidase inhibitory peptides.

### 3.5. Structural Characterization of EMO Peptide Fractions

#### 3.5.1. Surface Hydrophobicity of EMO Peptide Fractions

**Figure 5:**
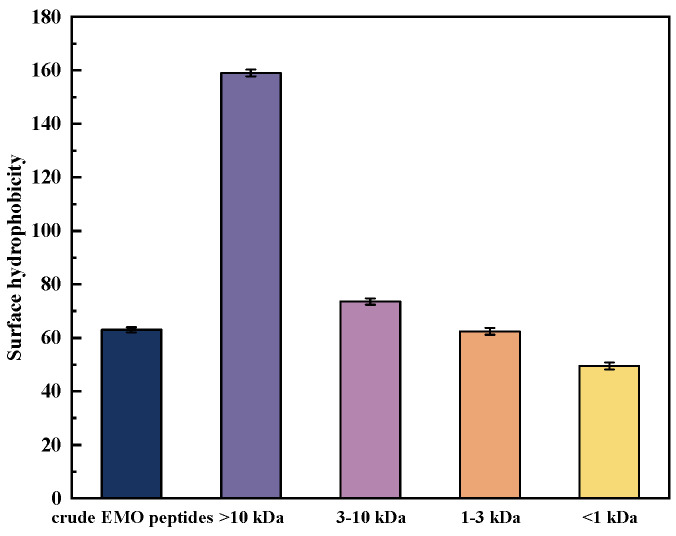
Surface hydrophobicity of EMO peptide fractions with different molecular-weight ranges (>10 kDa, 3–10 kDa, 1–3 kDa, and <1 kDa).

The surface hydrophobic characteristics of proteins and peptides are commonly evaluated using surface hydrophobicity (H_0_) as an index. As shown in Figure 5, ultrafiltration led to marked differences in H_0_ among the molecular weight fractions (*p* < 0.05). The >10 kDa fraction exhibited the highest H_0_ value (158.98), significantly higher than all other fractions, indicating that this fraction still contained a considerable proportion of hydrophobic macromolecular domains that had not been extensively hydrolyzed [39]. Within the ≤10 kDa range, H_0_ showed an overall decreasing trend with decreasing molecular weight, with the 3–10 kDa fraction being higher than the crude EMO peptides and the 1–3 kDa fraction, while the <1 kDa fraction displayed the lowest H_0_. This pattern of smaller peptides exhibiting weaker surface hydrophobicity can be understood as a consequence of progressive proteolysis: enzymatic hydrolysis cleaves originally continuous hydrophobic segments into shorter peptides and, at the same time, generates more terminal polar or charged groups, making these small peptides overall more hydrophilic and reducing the number of hydrophobic sites exposed at the molecular surface [40,41].

#### 3.5.2. Intrinsic Fluorescence Spectra of EMO Peptide Fractions

**Figure 6:**
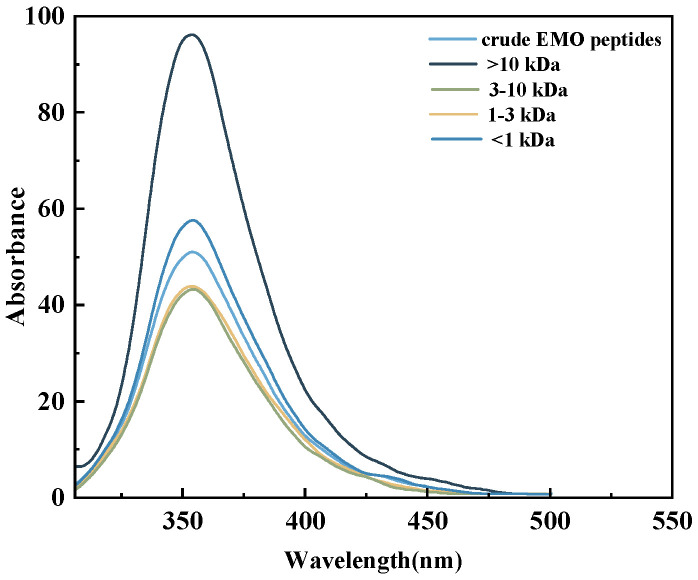
Fluorescence spectra of EMO peptide fractions with different molecular-weight ranges (>10 kDa, 3–10 kDa, 1–3 kDa, and <1 kDa).

Intrinsic fluorescence spectroscopy is widely used to probe the exposure of aromatic residues (Trp, Tyr, Phe) and thereby to monitor changes in protein and peptide tertiary structure [41]. As shown in Figure 6, all EMO peptide fractions exhibited similar emission profiles but different maximum emission wavelengths. The 3–10 kDa fraction showed the most red-shifted maximum (λ_max_ = 358.5 nm), compared with the >10 kDa (355.5 nm), 1–3 kDa (352.5 nm), <1 kDa (352.5 nm) fractions and the unfractionated crude hydrolysate (353.0 nm). Such a red shift is generally associated with an increase in the polarity of the microenvironment surrounding aromatic residues, indicating that enzymatic hydrolysis in the 3–10 kDa range partially disrupted the compact tertiary structure of EMO proteins and transferred more aromatic side chains from the hydrophobic core to the solvent-exposed surface [42,43]. This conformational rearrangement is likely to facilitate productive interactions between exposed aromatic residues and the active region of α-glucosidase [8], consistent with the highest inhibitory activity observed for the 3–10 kDa fraction.

With respect to fluorescence intensity, the >10 kDa fraction exhibited the strongest emission, followed by the crude EMO peptides, the <1 kDa and 1–3 kDa fractions, whereas the 3–10 kDa fraction showed the lowest fluorescence signal. Overall, the intrinsic fluorescence intensity of the fractions varied inversely with their α-glucosidase inhibitory activities: the most potent 3–10 kDa fraction displayed the weakest fluorescence. This pattern suggests that, in this fraction, more Trp residues are located in a polar aqueous environment and experience partial fluorescence quenching, which can occur when ligand binding and local structural loosening increase solvent exposure of Trp side chains [44]. These results support the view that solvent-exposed aromatic residues, especially Trp, play an important role in mediating the interaction between EMO peptides and α-glucosidase and thus contribute to the observed inhibitory activity.

#### 3.5.3. UV Absorption Spectra of EMO Peptide Fractions

**Figure 7:**
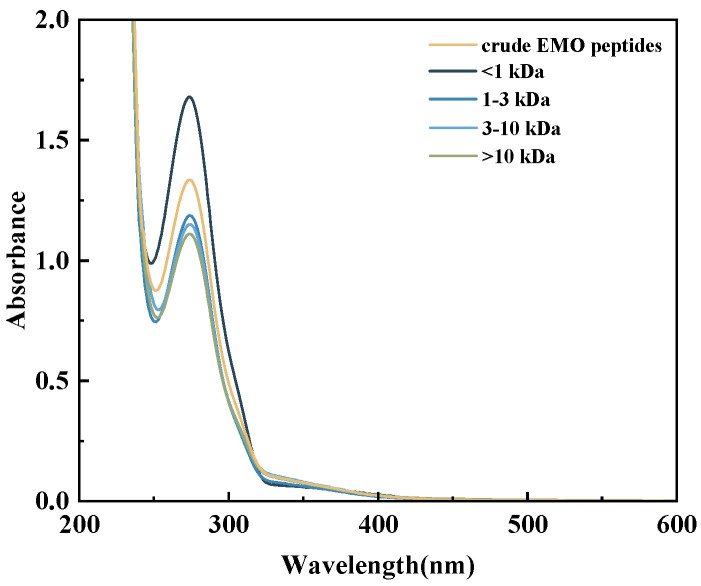
UV absorption spectra of EMO peptide fractions with different molecular-weight ranges (>10 kDa, 3–10 kDa, 1–3 kDa, and <1 kDa).

The UV absorption of peptides mainly arises from the n-π and π-π transitions of peptide bonds in the range of 200–230 nm and from the characteristic absorption of aromatic amino acid residues (Tyr, Trp and Phe) around 275–280 nm [45]. As shown in Figure 7, all EMO peptide fractions and the crude EMO peptides exhibited a strong absorption band between 200 and 230 nm and a distinct shoulder peak at approximately 275 nm. Compared with the crude EMO peptides and the >10 kDa fraction, the <1 kDa and 1–3 kDa fractions showed higher absorbance in both regions, revealing an overall trend that smaller molecular-weight fractions displayed stronger UV absorption. Previous work has demonstrated that the absorption near 275 nm is mainly attributable to Tyr and other aromatic residues. In the present study, progressive enzymatic hydrolysis is expected to cleave EMO protein macromolecules into medium and low molecular weight peptides, thereby generating more short peptide fragments containing aromatic residues and reducing the shielding and light scattering effects of large molecular aggregates on aromatic rings and peptide bonds [46]. Together with the intrinsic fluorescence and surface hydrophobicity data, these findings indicate that continuous hydrolysis not only modifies the molecular weight distribution, but also markedly alters the exposure and local microenvironment of aromatic residues in EMO peptides, which is closely related to their interaction with α-glucosidase. This interpretation is further supported by the molecular docking results, which showed that several peptides identified from the 3–10 kDa fraction could interact with catalytic or active pocket-related residues of α-glucosidase through hydrogen bonding, hydrophobic contacts, and other non-covalent interactions.

#### 3.5.4. Infrared Absorption Spectra of EMO Peptide Fractions

**Figure 8:**
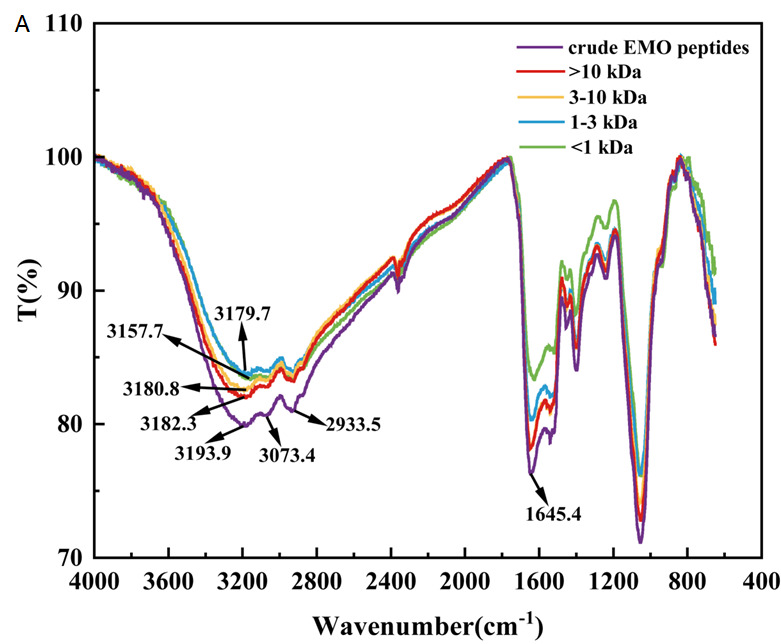

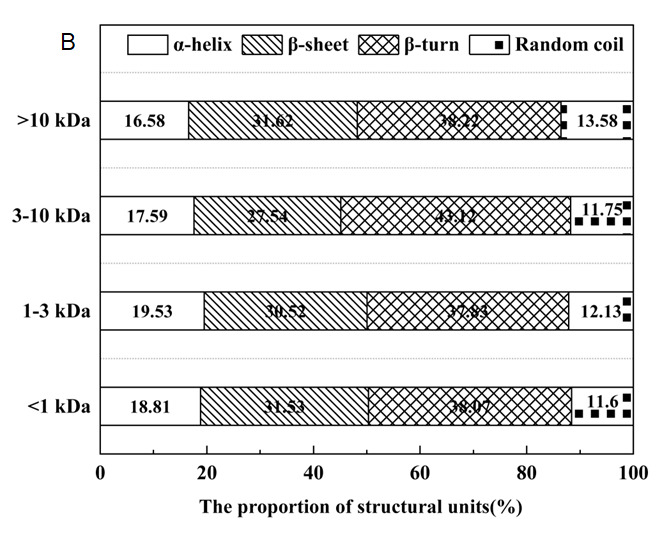
FTIR spectra (**A**) and secondary-structure composition (**B**) of EMO peptide fractions with different molecular-weight ranges (>10 kDa, 3–10 kDa, 1–3 kDa, and <1 kDa).

Fourier transform infrared (FTIR) spectroscopy is widely used to characterize the secondary structure of proteins and peptides, with particular attention to peptide-related bands in the 4000–400 cm^−1^ region, namely the amide A (3600–3000 cm^−1^), amide I (1700–1600 cm^−1^) and amide II (1600–1500 cm^−1^) bands [47]. As shown in Figure 8A, EMO peptide fractions with different molecular weights exhibited broadly similar spectral profiles: a broad amide A band around 3170–3200 cm^−1^, a C-H stretching band near 2933 cm^−1^, an amide I band at approximately 1645 cm^−1^ and an amide II band around 1530 cm^−1^. Minor differences were observed in the position and shape of the amide A band among fractions. The >10 kDa fraction showed a slightly broader amide A band, whereas the 3–10 kDa, 1–3 kDa and <1 kDa fractions displayed relatively sharper peaks, suggesting that the high molecular weight fraction still retains larger structural domains and a more complex hydrogen-bonding network, while the low molecular weight fractions consist of shorter peptide chains with more exposed hydrophilic residues and a more homogeneous hydrogen bonding environment.

To further quantify changes in secondary structure, the amide I region (1700–1600 cm^−1^) was deconvoluted and fitted with Gaussian components to resolve α-helix, β-sheet, β-turn and random coil elements (Figure 8B). Overall, all four molecular-weight fractions were dominated by β-sheet and β-turn structures, whereas α-helix and random coil contents were relatively low. Compared with the >10 kDa fraction, the 3–10 kDa and <1 kDa fractions showed slightly higher proportions of β-turn and slightly lower random coil contents, with the 1–3 kDa fraction falling in between. The α-helix content varied only modestly within the range of about 16–20%. The somewhat higher β-turn content in the 3–10 kDa fraction is consistent with its stronger α-glucosidase inhibitory activity, suggesting that a moderate enrichment of β-turns in this fraction may be related to a more favorable conformation for enzyme binding and inhibition [33,34].

### 3.6. Stability of EMO Peptides During Processing and Simulated Digestion

**Figure 9:**
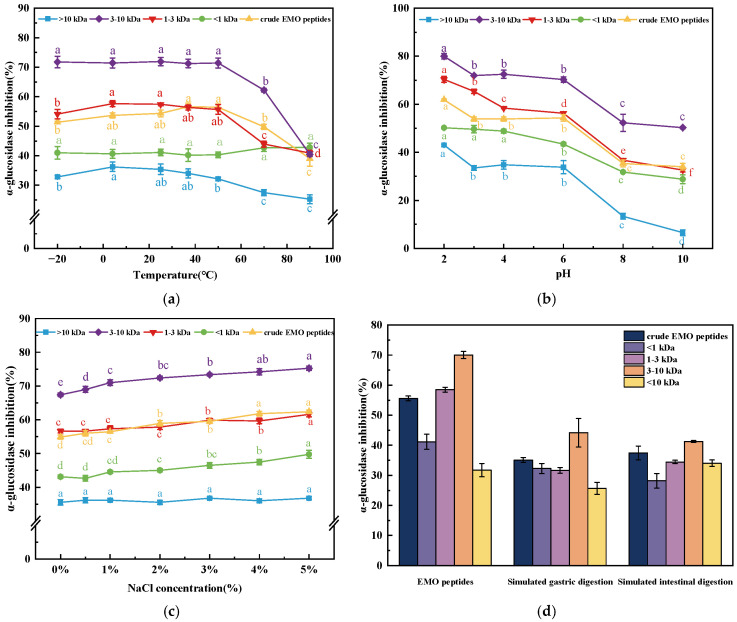
Stability of EMO peptide fractions during processing and simulated digestion. (**a**) Temperature; (**b**) pH; (**c**) NaCl concentration; (**d**) simulated gastrointestinal digestion. Different lowercase letters (**a**–**d**) on the same curve indicate significant differences among different treatment conditions within the same peptide fraction (*p* < 0.05).

The α-glucosidase inhibitory stability of EMO peptide fractions under different processing and digestion conditions is shown in Figure 9. Across the temperature range −20 to 50 °C, the inhibitory activities of the >10 kDa, 3–10 kDa, 1–3 kDa, <1 kDa fractions and crude EMO peptides did not change significantly (*p* > 0.05), indicating that low to moderate temperatures have little impact on their bioactivity during chilled storage or mild processing. When the temperature was raised to 50–100 °C, the activities of the >10 kDa, 3–10 kDa and 1–3 kDa fractions decreased compared with their initial values, whereas the <1 kDa fraction remained essentially stable (Figure 9a). This pattern suggests that high temperatures are detrimental to medium and high molecular weight peptides, which are more prone to heat-induced unfolding, aggregation or peptide-bond cleavage, but less so to low molecular weight peptides whose shorter chains confer greater conformational flexibility and heat tolerance [48].

In the pH experiment, all four fractions and crude EMO peptides showed increased inhibitory activity at pH 2–6, with a maximum at pH 2 (Figure 9b), indicating good acid resistance and an overall preference for mildly to strongly acidic conditions. This pattern is consistent with Li et al. (2023), who reported enhanced α-glucosidase inhibition of the hypoglycemic peptide YPVEPF from cheese whey hydrolysate at acidic pH [49]. The enhancement observed at low pH may reflect a more favorable protonation state of key ionizable residues, which strengthens electrostatic interactions with the α-glucosidase active site, whereas excessive deprotonation under alkaline conditions can disrupt these interactions. Accordingly, when the pH was raised to 8.0 and 10.0, the activities of the >10 kDa, 3–10 kDa, 1–3 kDa, <1 kDa fractions and crude EMO peptides declined to 6.60%, 50.27%, 32.62%, 28.79% and 34.02%, respectively, showing that alkaline conditions impair the α-glucosidase inhibitory capacity of EMO peptides and that, from a processing and storage perspective, acidic matrices are more suitable to preserve their functionality.

Varying NaCl concentration from 0 to 5% led to a gradual increase in inhibitory activity in all four fractions and crude EMO peptides (Figure 9c), suggesting that typical salt levels used in foods do not adversely affect EMO peptide function and that moderate ionic strength may even stabilize peptide conformation and facilitate peptide and enzyme recognition rather than screening key interactions. In vitro-simulated gastrointestinal digestion further revealed that, after sequential pepsin and trypsin treatment, the >10 kDa fraction exhibited increased activity, whereas the 3–10 kDa, 1–3 kDa, <1 kDa fractions and crude EMO peptides showed varying degrees of decline (Figure 9d). This likely reflects a dynamic balance between the generation of new bioactive fragments from large precursors and the over-hydrolysis of smaller peptides into inactive or weakly active sequences. Nevertheless, the 3–10 kDa fraction maintained the highest activity at the end of the gastric and intestinal phases, with inhibition rates of 44.19% and 41.27%, respectively. Taken together, these findings indicate that EMO peptides display good processing stability under acidic conditions and typical NaCl levels, and retain appreciable α-glucosidase inhibitory activity after simulated gastrointestinal digestion, highlighting their overall suitability for incorporation into thermally processed, acidified functional foods.

The present results indicate that the hydrolysates prepared from EMO meal by enzymatic hydrolysis possess a certain degree of α-glucosidase inhibitory activity, show good safety, and retain part of their activity under acidic conditions, at common salt concentrations, and after simulated gastrointestinal digestion. These findings suggest that EMO hydrolysates have the basis for further development as food-derived α-glucosidase inhibitory ingredients and may also serve as potential active materials for functional foods. As an oil processing by-product, EMO meal has long been underutilized as a protein resource. The present study demonstrates that it can be converted by enzymatic hydrolysis into peptide products with glucose-lowering activity, providing a new route for the value-added utilization of this by-product, which is also consistent with the goals of sustainable use and waste reduction. From a development perspective, crude peptides and ultrafiltration fractions do not simply represent a better or worse relationship, but rather meet different needs. Crude peptides, with their simpler preparation process and relatively higher yield, may be more suitable as a basis for subsequent functional food ingredient development and food system evaluation. In contrast, the main value of ultrafiltration lies in narrowing the molecular weight distribution and enriching peptide fractions that contribute more strongly to the overall activity, thereby facilitating activity localization, mechanistic investigation, and the further screening of key functional peptides. In the present study, the 3–10 kDa fraction showed higher α-glucosidase inhibitory activity, suggesting that this fraction may contain key peptide groups that contribute substantially to the overall activity of the crude EMO hydrolysate. Overall, EMO hydrolysates may be developed either as crude peptide ingredients for basic functional food applications or as enriched active fractions for further characterization and more targeted development. Further studies are still needed to evaluate their practical applicability in real food matrices, as well as their storage stability and in vivo effects.

### 3.7. Inhibitory Mechanism of EMO Peptides Against α-Glucosidase

To further investigate the inhibitory mechanism of EMO peptides against α-glucosidase, we focused on the 3–10 kDa fraction, which showed the highest inhibitory activity. Its peptide sequences were identified by LC-MS/MS, and the interaction mechanism with α-glucosidase was analyzed using enzyme inhibition kinetics, fluorescence quenching, circular dichroism and molecular docking.

#### 3.7.1. 3–10 kDa Peptide Fraction Sequence Analysis

**Figure 10:**
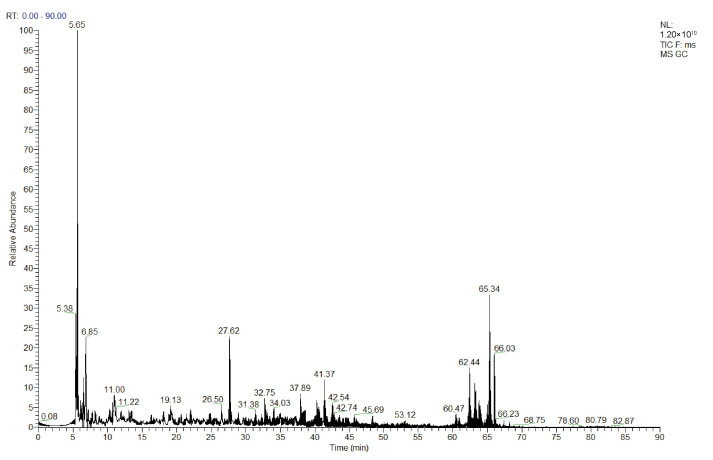
Total ion chromatogram of EMO peptides.

LC-MS/MS was used to characterize the peptide sequences present in the 3–10 kDa EMO fraction, and the corresponding total ion chromatogram is shown in Figure 10. By combining de novo sequencing with database searching, 34 distinct peptides were identified, with theoretical molecular weights ranging from 957 to 3765 Da. The theoretical molecular weight range of these peptides does not match the nominal 3–10 kDa range assigned to this ultrafiltration fraction. A discrepancy that is commonly reported when using MWCO membranes for coarse fractionation. The molecular weight cut-off reflects an approximate retention threshold rather than a strict upper and lower boundary, and the actual separation profile is continuous rather than discrete. Consequently, some relatively small peptides can still be retained in the 3–10 kDa retentate, leading to partial overlap between adjacent fractions and an apparent mismatch between the nominal fraction label and the molecular weights of the identified peptides.

On this basis, the amino acid sequences of the 34 peptides were further analyzed, and their overall composition is summarized in Table 3. In total, 20 different amino acids were represented, with glycine, threonine, serine, leucine, tyrosine and proline occurring at relatively high frequencies. Notably, hydrophobic aliphatic residues such as glycine, valine, proline, leucine and alanine together accounted for 41.8%. Previous studies have shown that such hydrophobic residues facilitate the insertion of small inhibitors into the hydrophobic pocket of carbohydrate-digesting enzymes and stabilize binding through hydrophobic contacts and hydrogen bonds, thereby contributing to glucose-lowering effects [50]. Leucine, in particular, has also been implicated in the regulation of glycemic control in vivo. In addition, basic amino acids such as arginine and sulphur-containing residues such as methionine represented 8.5% of the total composition. Consistent with this, Mohd Rodhi et al. (2023) reported that specific charged and aromatic functional groups at the inhibitor interface can interact with residues around the enzyme active site, block substrate access and reinforce inhibitory potency [51]. Taken together, the amino acid composition of these 34 peptides suggests a favorable structural basis for forming stable peptide-enzyme interactions, consistent with the high α-glucosidase inhibitory activity observed for this fraction.

#### 3.7.2. Kinetic Analysis of α-Glucosidase Inhibition by the 3–10 kDa Peptide Fraction

**Figure 11:**
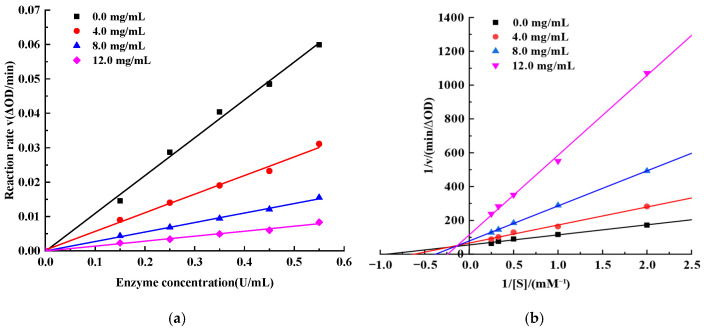
Kinetic analysis of α-glucosidase inhibition by the 3–10 kDa EMO peptide fraction. (**a**) Enzyme concentration-velocity curve. (**b**) Lineweaver–Burk plots at different inhibitor concentrations (0, 4.0, 8.0 and 12.0 mg/mL).

In general, enzyme inhibitors can interact with the catalytic site either through covalent bonding or via non-covalent forces. The former usually leads to irreversible loss of enzyme activity and is classified as irreversible inhibition, whereas the latter is typically reversible, since the conformational changes induced in the enzyme can be restored once the inhibitor is removed [52]. To further elucidate the mode of action of the 3–10 kDa EMO peptide fraction on α-glucosidase, the relationship between enzyme concentration and initial reaction rate was examined at a fixed substrate concentration (Figure 11a). As the α-glucosidase concentration increased, the initial reaction rate increased linearly; at the same enzyme concentration, higher levels of the 3–10 kDa peptide fraction resulted in smaller slopes of the fitted lines, indicating lower reaction rates. All regression lines passed through the origin, a kinetic feature that is consistent with reversible inhibition. Tang et al. (2019) also observed that the regression lines of enzyme concentration versus reaction rate at different levels of salvianolic acid C intersected at the origin, and accordingly classified it as a reversible inhibitor [50].

The inhibition type of α-glucosidase by the 3–10 kDa peptide fraction was determined using Lineweaver–Burk double reciprocal plots. As shown in Figure 11b, increasing the peptide concentration from 0.0 to 12.0 mg/mL led to a gradual decrease in V_max_ from 18.19 to 8.78 μM/min, while K_m_ increased from 1.08 to 4.14 mM, and the fitted lines at different inhibitor levels intersected in the second quadrant. This pattern is characteristic of a mixed-type inhibition mechanism [53].

#### 3.7.3. Fluorescence Quenching of α-Glucosidase by the 3–10 kDa Peptide Fraction

**Figure 12:**
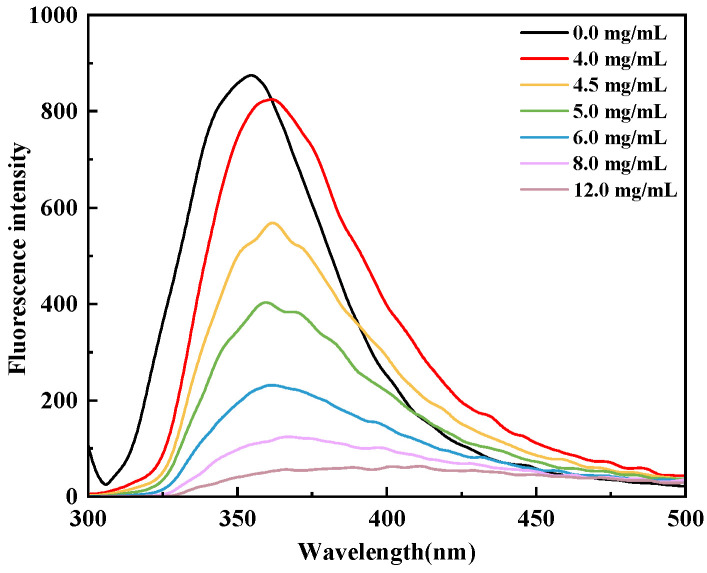
Fluorescence quenching spectra of α-glucosidase in the presence of different concentrations of the 3–10 kDa EMO peptide fraction.

The 3–10 kDa peptide fraction induced pronounced quenching of the intrinsic fluorescence of α-glucosidase, indicating direct interaction between the peptides and the enzyme and suggesting associated conformational changes. Intrinsic fluorescence spectroscopy is widely applied to monitor alterations in the microenvironment of aromatic residues during protein-ligand binding [42]. As shown in Figure 12, at an excitation wavelength of 280 nm, native α-glucosidase exhibited a maximum emission peak at 356 nm. Upon gradual addition of the 3–10 kDa peptide fraction, the fluorescence intensity of α-glucosidase decreased progressively in a concentration dependent manner, and the maximum emission wavelength shifted towards longer wavelengths. This red shift implies an increase in the polarity of the microenvironment surrounding tyrosine and tryptophan residues and is consistent with local conformational adjustments of the enzyme [54]. These spectral changes indicate that the 3–10 kDa peptide fraction binds to α-glucosidase and perturbs its tertiary structure, providing molecular-level support for the inhibitory effects observed in the kinetic analysis.

#### 3.7.4. Circular Dichroism Analysis

**Figure 13:**
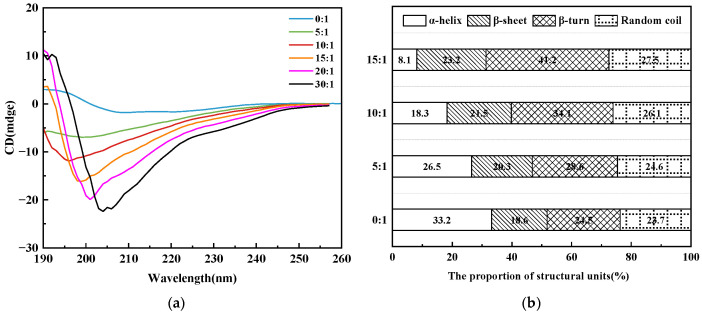
CD spectra (**a**) and secondary-structure composition (**b**) of α-glucosidase in the presence of the 3–10 kDa EMO peptide fraction.

Circular dichroism (CD) spectroscopy was used to characterize the secondary structure of α-glucosidase and to assess the structural changes induced by the 3–10 kDa peptide fraction. As shown in Figure 13a, native α-glucosidase exhibited two pronounced negative minima at 209 nm and 220 nm, which are consistent with a secondary structure rich in α-helices [55]. Upon addition of the 3–10 kDa peptide fraction, these characteristic minima gradually shifted towards lower wavelengths and their ellipticity decreased as the peptide concentration increased. This concentration dependent attenuation of the α-helical signals indicates that binding of the peptide fraction perturbs the secondary structure of α-glucosidase.

To quantify these changes, the CD spectra were deconvoluted using CDNN (version 2.1) software, and the relative contents of different secondary structural elements in α-glucosidase and in the α-glucosidase peptide complexes were estimated (Figure 13b). With increasing ratios of the 3–10 kDa peptide fraction to α-glucosidase, the proportion of α-helix decreased from 33.20% to 8.10%, whereas the contents of β-sheet, β-turn and random coil increased accordingly [56]. These shifts suggest that peptide binding induces partial unfolding of helical regions and a transition from a more compact to a more relaxed conformation. In line with the kinetic and fluorescence results, these structural data support the hypothesis that the 3–10 kDa peptide fraction disrupts the hydrogen-bond network stabilizing α-helical segments in α-glucosidase, increases the exposure of polar regions and alters the geometry of the substrate binding site, thereby impairing substrate recognition and contributing to the inhibitory effect on α-glucosidase activity [57].

#### 3.7.5. Molecular Docking

**Figure 14:**
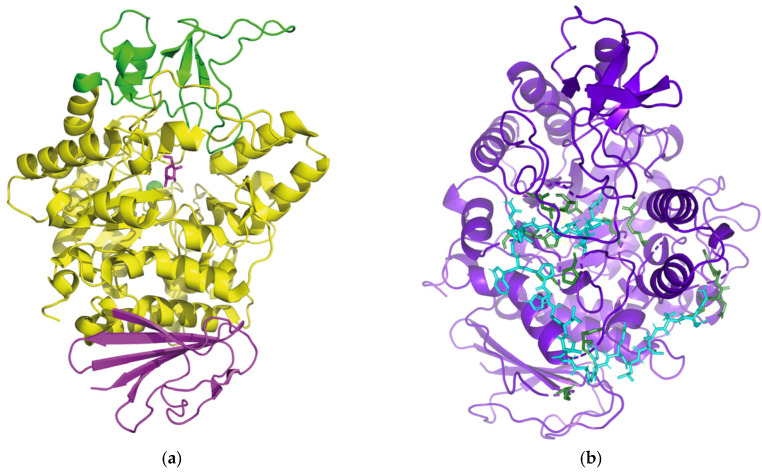

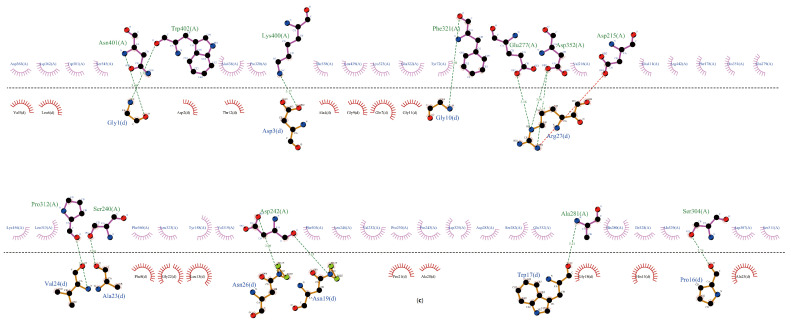
Structure of α-glucosidase and its interaction with an EMO peptide (sequence 14). (**a**) Stereoview of the α-glucosidase structure, highlighting domain A (yellow), domain B (green), and domain C (purple). (**b**) Three-dimensional binding mode of peptide 14 with α-glucosidase. (**c**) Two-dimensional interaction diagram of peptide 14 with α-glucosidase.

Molecular docking was employed to gain further insight into the interaction mechanism between the 3–10 kDa peptide fraction (ligands) and α-glucosidase (receptor). This approach allows the identification of key non-covalent interactions, including hydrogen bonding, hydrophobic contacts and electrostatic interactions, while providing an estimate of binding affinity through binding energy scores. In general, a binding energy below −5.0 kcal/mol is considered to indicate a favorable interaction, with more negative values reflecting a more stable complex [58]. The 34 peptide sequences identified from the 3–10 kDa fraction by LC-MS/MS were docked to α-glucosidase using AutoDock Vina. Among these, 21 peptides exhibited binding energies lower than −5.0 kcal/mol, with values ranging from −5.9 to −9.3 kcal/mol. The detailed binding energies, interaction modes and binding sites are summarized in Table 4. The α-glucosidase model used in this study (from Saccharomyces cerevisiae, PDB: 3A4A) comprises three structural domains (A, B and C; Figure 14a). Domain A (residues 1–113 and 190–512) contains three catalytic acidic residues, D215, E277 and D352, which form the core of the catalytic site of α-glucosidase [59]. Docking results showed that peptides 1, 2, 3, 5, 12, 14, 15 and 20 each interacted with at least one of these catalytic residues, and all displayed relatively low binding energies (≤−7.0 kcal/mol), with peptide 14 exhibiting the lowest value (−9.3 kcal/mol). The interaction patterns with the three catalytic residues differed among peptides. At D352, peptides 1, 3 and 12 mainly engaged through hydrophobic contacts, whereas peptides 2, 14 and 20 formed hydrogen bonds, and peptide 15 formed a salt bridge. At D215, peptide 5 interacted predominantly via hydrophobic contacts, while peptide 14 formed a salt bridge. At E277, peptides 2, 3, 14 and 15 established hydrogen bonds, whereas peptides 5 and 12 interacted mainly through hydrophobic contacts. Notably, peptide 14 was the only sequence that simultaneously interacted with all three catalytic residues (D215, E277 and D352). These findings suggest that whether a peptide engages catalytic residues, how many such residues are involved, and the nature of the interactions (hydrogen bonding, hydrophobic contacts or salt bridges) together influence the extent of α-glucosidase inhibition. They also support the conclusion from kinetic analysis that the mixed-type inhibition of the 3–10 kDa peptide fraction contains a strong competitive component, with peptides preferentially occupying the active site pocket and thereby sterically hindering substrate access.

Previous studies have also identified D69, R442 and E411 as functionally important residues in α-glucosidase [60]. In the present docking analysis, peptides 1, 2, 4, 5, 9, 10, 11, 12, 13, 14 and 15 each interacted with at least one of these residues, which may contribute to local conformational adjustments and further reductions in enzymatic activity. In addition, residues Y158 and H280 and the loop region spanning residues 310–315, located at the entrance of the active-site pocket, have been implicated in modulating substrate access [59]. Peptides 1–16 and 20 were all predicted to interact with this entrance region, suggesting that they may physically obstruct substrate binding to the catalytic site. By contrast, peptides 17, 18, 19 and 21 did not interact with the key catalytic residues described above and instead bound to residues surrounding the active site [61]. This is consistent with their relatively higher binding energies and presumably weaker inhibitory potential.

Taken together, these results indicate that peptide 14 (sequence GDDAVLQFGGGTLGHPWGNAPGAVANR) is the most promising inhibitory peptide among the identified 3–10 kDa sequences, as it forms interactions with multiple catalytically relevant residues, including D215, E277 and D352. Visualization of the docking pose of peptide 14 using PyMOL and LIGPLOT (Figure 14b,c) showed that residue R27 of the peptide forms hydrogen bonds with E277 and D352 and engages in electrostatic interactions with D215, suggesting a key contribution of the arginine side chain to the inhibition of α-glucosidase activity. Hydrogen bonding has frequently been reported as an important determinant of α-glucosidase inhibition, with both the number and length of hydrogen bonds proposed to affect binding stability and inhibitory potency [10,59]. In this study, however, peptide 21 formed more hydrogen bonds with shorter average bond lengths than peptide 14, yet displayed a higher (less favorable) binding energy and weaker predicted inhibitory potential. This discrepancy indicates that hydrogen bond number and distance alone are not sufficient to explain the inhibitory strength of peptides. Instead, the specific involvement of key catalytic residues, the spatial location of the binding site within or around the active pocket, and the combined contribution of multiple interaction types appear to play a more decisive role in determining the overall inhibitory effect of the 3–10 kDa peptide fraction on α-glucosidase.

As is well known, acarbose, as a classical reversible competitive inhibitor of intestinal α-glucosidase, differs markedly from peptides in both its mode of action and binding sites, mainly because acarbose is a small pseudo-oligosaccharide inhibitor, whereas peptides possess larger molecular sizes and greater conformational flexibility. Molecular docking based on the 3A4A model showed that acarbose was mainly located in the catalytic pocket of α-glucosidase and its adjacent entrance region, and formed a hydrogen bonding network with residues such as Asp215, His351, Asp352, and Arg442, indicating that acarbose exerts its inhibitory effect primarily by occupying the active region and interacting with catalytically relevant residues [62]. EMO-derived peptides were likewise found to interact with both the catalytic region and the pocket entry region, suggesting a certain similarity to acarbose in terms of acting on enzyme activity-related regions. However, the marked differences in molecular size and conformational characteristics between peptides and acarbose also indicate that their inhibitory mechanisms toward α-glucosidase are not entirely identical. Based on these differences, acarbose and EMO-derived peptides may also differ in their modes of action and in the side effects they may potentially induce. Whether EMO-derived peptides would produce intestinal responses similar to those of acarbose still requires further verification through cell-based assays, gastrointestinal digestion stability studies, and animal experiments.

## 4. Conclusions

In this study, *Elaeagnus mollis* oilseed meal (EMO) was evaluated as a novel source of food-derived α-glucosidase inhibitory peptides. Hydrolysis with 3.350 acidic protease followed by ultrafiltration produced a 3–10 kDa peptide fraction with the strongest inhibitory activity. This fraction retained substantial inhibitory potency over a relevant range of pH, temperature, NaCl concentrations and simulated gastrointestinal conditions and showed no detectable cytotoxicity within the tested concentration range, indicating good functional stability and safety in vitro. Kinetic analysis showed a mixed-type inhibition pattern, indicating that EMO peptides can interact with both the free enzyme and the enzyme–substrate complex and tend to occupy the catalytic site. Fluorescence and circular dichroism spectroscopy further demonstrated that peptide binding alters the microenvironment of aromatic residues and the secondary structure of α-glucosidase, consistent with local conformational rearrangements of the enzyme. LC-MS/MS sequencing combined with molecular docking showed that peptides from this fraction form stable complexes with key catalytic and neighboring residues through hydrogen bonding, hydrophobic contacts and electrostatic interactions. Taken together, these results indicate that EMO-derived peptides enriched in hydrophobic and acidic residues are promising α-glucosidase inhibitors and provide sequence-level and mechanistic information to support their further evaluation in cell, animal and model food systems.

## Figures and Tables

**Table 1:** Optimum conditions for protease activity.

Protease	Optimum pH	Optimum Temperature (°C)
Longda acidic protease (700,000 U/g)	3.0	50
Longda acidic protease (800,000 U/g)	3.0	50
Longda acidic protease (35,000 U/g)	3.0	50
3.350 acidic protease (43,000 U/g)	3.0	50
537 acidic protease (200,000 U/g)	3.0	50
537 acidic protease (20,000 U/g)	3.0	50
Solarbio acidic protease (50,000 U/g)	3.0	50
Pepsin (3000 U/mg)	2.0	37
Bacillus neutral protease (195,000 U/g)	7.0	35
Solarbio neutral protease (50,000 U/g)	7.0	50
Amano neutral protease (55,000 U/g)	6.0	50
9530 neutral protease (11,000 U/g)	7.0	65
Flavour protease (100,000 U/g)	7.0	53
Bromelain (850,000 U/g)	7.0	55

**Table 2:** Screening of proteases for the preparation of α-glucosidase inhibitory peptides from EMO meal. Different lowercase letters (a–g) in the same column indicate significant differences (*p* < 0.05).

Protease	α-Glucosidase Inhibition (%)	Peptide Yield (%)
Longda acidic protease (700,000 U/g)	29.99 ± 3.17 ^cd^	46.85 ± 2.14 ^c^
Longda acidic protease (800,000 U/g)	30.68 ± 1.69 ^cd^	52.10 ± 3.38 ^b^
Longda acidic protease (35,000 U/g)	32.08 ± 3.08 ^c^	56.52 ± 2.10 ^a^
3.350 acidic protease (43,000 U/g)	43.38 ± 1.69 ^a^	58.68 ± 2.13 ^a^
537 acidic protease (200,000 U/g)	40.31 ± 1.28 ^ab^	47.60 ± 3.09 ^c^
537 acidic protease (20,000 U/g)	36.96 ± 3.80 ^b^	51.18 ± 0.94 ^b^
Solarbio acidic protease (50,000 U/g)	24.96 ± 3.41 ^d^	49.60 ± 0.38 ^bc^
Pepsin (3000 U/mg)	31.38 ± 1.51 ^cd^	36.35 ± 2.25 ^d^
Bacillus neutral protease (195,000 U/g)	11.72 ± 1.92 ^f^	22.00 ± 1.17 ^f^
Solarbio neutral protease (50,000 U/g)	24.55 ± 0.64 ^d^	18.50 ± 0.84 ^g^
Amano neutral protease (55,000 U/g)	36.40 ± 2.22 ^b^	18.48 ± 0.60 ^g^
9530 neutral protease (11,000 U/g)	34.73 ± 1.51 ^bc^	25.21 ± 1.35 ^e^
Flavour protease (100,000 U/g)	28.73 ± 1.34 ^cd^	19.33 ± 1.42 ^fg^
Bromelain (850,000 U/g)	27.62 ± 2.93 ^d^	26.78 ± 0.10 ^e^

**Table 3:** Amino acid composition of peptides in the 3–10 kDa fraction identified by LC-MS/MS.

Amino Acid	Number of Residues	Proportion (%)
Glutamine	20	3.6%
Phenylalanine	22	4.0%
Glycine	92	16.8%
Threonine	53	9.7%
Leucine	40	7.3%
Histidine	18	3.3%
Proline	33	6.0%
Tryptophan	14	2.6%
Valine	29	5.3%
Isoleucine	5	0.9%
Serine	45	8.2%
Glutamic acid	18	3.3%
Aspartic acid	37	6.8%
Asparagine	24	4.4%
Methionine	6	1.1%
Arginine	10	1.8%
Lysine	10	1.8%
Alanine	30	5.5%
Tyrosine	38	6.9%
Cysteine	3	0.5%

**Table 4:** Molecular docking results.

No.	Sequence	MW (Da)	Binding Site on α-Glucosidase			Binding Energy (kcal/mol)
			Hydrophobic	H-Bonds	Salt Bridges	
1	FGGGTLGHPWGNAP	1366.6148	T310, F312, V319, D325, G309, V308, I328, D307, A281, H280, S282, D242, P243, L246, K156, F314, F159, R442, D352, E411, F178, F303	S304, E332, S240, P312, D283, Y158, Q279		−8.9
2	QFGGGTLGHPWGNA	1397.6476	S282, I328, A329, A281, N302, H280, V308, D325, G309, F321, R442, Y158, S157, K156, D307, L246, P243, V232	D283, E332, S304, D352, E277, R315, P312, D242, S240	E332	−8.9
3	DYLTGGFTANTTLAH	1580.7471	S240, V319, T310, S311, D307, V308, Q279, D352, R315, S282, N302, A329, I328, F314, L313	D242, P312, E277, H280, A281, S304, E332, Y158, D325	D325	−8.6
4	LGPTGVGKTE	957.5131	H280, D242, K156, S240, L313, P312, S311, F314, R315, Q279, F159, R442, F178, V308, D325, G309, V319	E332, S304, D307, T310, Y158, E411		−7.3
5	LFGGAGVGKTVL	1117.6495	H280, S240, P312, E411, V308, D307, F303, T306, D352, I328, A329, D325, R442, V216, F178, D215, Y72, F159, E277, H112, D69, A281	D242, Y158, R315, Q353, E332		−8.0
6	LPGDNVGFNVK	1158.6033	S282, A329, I328, D307, F314, S311, R315, L246, D242, S157, K156, D325, T310, V308	A281, E332, S304, H280, P312, Y158, S240		−7.5
7	QFGGGTLGHPW	1155.5461	H280, S311, P312, F303, R315, K156, Y158, V232, P243, I328, A329, V308, A281, S282	T310, S240, D242, L246, T285, N247, E332, S304	D307	−9.0
8	GPTGLTTEVK	1001.5393	P320, V308, V319, E411, Y316, R315, F314, L313, Y158, S240, F303, A329, S304, I328, D307, T310	S241, K156, D242, Q279, D325, H280, P312		−7.9
9	APMFVVGVNEK	1189.6165	L246, Q279, F314, Y316, E411, Y158, S240, K156, S157, S241, L313, P312, D307, V308, P243, E332, A281, S282	H280, D242, R315, N415, T310,		−7.5
10	QFGGGTLGHPWGN	1326.6105	V303, A329, D325, I328, F321, V319, E332, S311, R315, F314, D307, H280, A281, N247, S282, L246, S240, S157, K156, Y158, E411, Q279	S304, P320, P312, D242, L313		−8.5
11	DNGLLLHIH	1030.5559	P243, S304, S311, L313, P312, D242, N302, S241, K156, S240, S157, R315, F303, F314, F178, Y316, Y158, F159	S282, L246, A281, E332, H280, D307, E411		−7.9
12	QFGGGTLGHPWGNAP	1494.7004	Y158, F303, D352, Q279, E277, R315, D307, H280, L313, V232, P243, S240, E332, A281, S282, V308, P320, A329, D325, I328, T285, N247, L246	R442, T310, S304, P312		−8.6
13	GDDAVLQFGGGTLGHPWGNAPGAVANRVA	2803.3789	W581, F360, F543, G361, L439, R359, T358, S364, K524, S544, E322, P320, D325, S304, A329, V319, V308, G309, S311, H280, N247, A281, P230, D242, V232, L313, P243, K156, F159, G160, F314, E411	D362, K523, F321, E332, T310, D307, P312, T285, S282, R287, E284, D283, S240, L246, Y158, N415, R315	D363, D307	−8.4
14	GDDAVLQFGGGTLGHPWGNAPGAVANR	2633.2734	D363, D362, W581, S545, A438, P320, T358, L439, K523, E322, Y72, V216, E411, R442, F178, H351, Q279, K156, L313, F360, L323, Y158, V319, F303, L246, V232, P230, P243, D325, D283, S282, E332, H280, I328, A329, D307, S311	N401, W402, K400, F321, E277, D352, P312, S240, D242, A281, S304	D215	−9.3
15	VASGGIHVWH	1061.5406	D283, S282, F303, F178, E411, R442, R315, S304, T310, Y158, P312, K156, S240, V232	E332, A281, N302, E277, H280	D352 D307	−8.9
16	EYPEYEDENPYLYQEEHFDTRLK	3006.3193	P249, V308, V319, D325, S311, A329, V232, F314, D242, Q239, L313, E332, I328, D283, P243, L246, L288	T310, P312, D307, S304, H280, R315, A281, S240, S282, N247, E284, T285, H252	D307 K156	−6.1
17	KDSSATKLSGYTWDLSYGDGSSASGDVYDR	3229.4321	D34, A465, Y470, P467, W468, Q67, V404, Y416, E405, G161, G160, S162, R176, N414, M70, R413, Y407, E408, C179, T165, D167, S115, E116, T181, W81, S65	K466, N417, D68, S180, K169, E168, F166, Y174, R124, T83	E421	−5.9
18	SSSATKLSGYTWDLSYGDGSSASGDVYRD	3031.3318	Y407, E405, Y416, N417, V404, M70, Q67, W468, E408, G160, N414, R176, K148, I150, P151, W238, E421, S162, G161, T165, W164, F173, P149, T181, E168, K169, K121, E116	D68, R413, S180, E146, D144, F166, S115	R124	−6.4
19	YPANSGDVDEYEDENPYLYQEEHFDRT	3294.3535	T165, W81, K406, E408, W468, T181, D64, P66, V410, C179, F166, F173, P149, K148, W164, G161, Q67, W36, Y416, E421, K420	T83, Y407, Y470, E405, S115, R124, Y174, E116, D68, S180, S162, E168, R176, R413, N414, N417	R413	−6.3
20	YTGGGQGGTYGTTTDTDYVNWYGTTTGTGGTYDN	3501.4392	W581, K523, S544, K524, E322, L439, F321, W326, F360, L323, P320, D325, I328, E428, R359, K234, N235, E429, D233, H423, F314, V319, P312, H280, E332, N302, D307, A281, V308, R315, L246, P243, R442, E411, F303, Q279	S545, E435, L318, K432, T358, N317, S311, L313, S304, S240, D352, Y158		−7.2
21	TNYGQNTYGTDSPHGYGGTHSYSATTGTNTYD	3387.3823	E421, G161, E405, G160, S162, I412, V404, Y416, F469, M70, T181, F166, F173, W164, P151, D34, W468, P149, W238, T83, K466, W81, V410	K402, N417, N414, Y407, Y470, E408, R413, R176, S180, Y174, T165, E168, K148, P82, P467, W36, D68	R413	−6.1

## Data Availability

The original contributions presented in this study are included in the article/Supplementary Materials. Further inquiries can be directed to the corresponding author.

## Supplementary Materials

The following supporting information can be downloaded at: https://www.mdpi.com/article/10.3390/foods15081323/s1, Supplementary Material S1: Single-factor experiments and orthogonal optimization of the hydrolysis conditions for EMO peptides with α-glucosidase inhibitory activity.
