# Supplementary material for: Preparation and Mechanistic Characterization of α-Glucosidase Inhibitory Peptides from Elaeagnus mollis Oilseed Meal
Source: Foods. 2026 Apr 10;15(8):1323. doi: 10.3390/foods15081323 (PMC13115018; doi:10.3390/foods15081323)
Supplement: Supplementary file 1 [file foods-15-01323-s001.zip › foods-4207069-supplementary.pdf]

Supplementary Material S1. Single-factor experiments and orthogonal optimization of the hydrolysis conditions for EMO peptides with α-glucosidase inhibitory activity

S1. Single-factor experiments and orthogonal optimization of hydrolysis conditions

S1.1 Effect of substrate concentration

The effect of substrate concentration on peptide yield and α-glucosidase inhibition was investigated at pH 3, 50 °C, an enzyme dosage of 5000 U/g, and a hydrolysis time of 1 h (Fig. S1). When the substrate concentration increased from 2% to 16%, both peptide yield and α-glucosidase inhibition first increased and then decreased. The highest peptide yield (70.39%) and α-glucosidase inhibition (45.39%) were obtained at 6%. Therefore, a substrate concentration of 6% was selected for subsequent experiments.

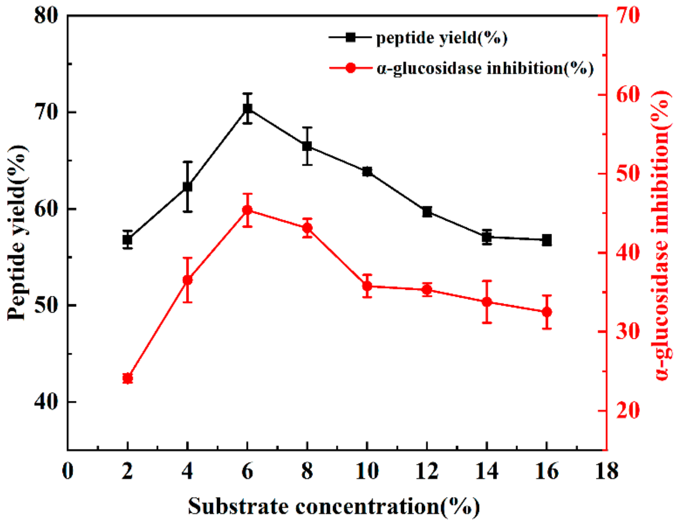

Fig. S1. Effect of substrate concentration on peptide yield and α-glucosidase inhibition.

S1.2 Effect of enzyme dosage

The effect of enzyme dosage on peptide yield and α-glucosidase inhibition was evaluated at a substrate concentration of 6%, pH 3, 50 °C, and a hydrolysis time of 1 h (Fig. S2). In the range of 1000-9000 U/g, peptide yield increased with increasing enzyme dosage and approached a plateau at 5000 U/g (69.40%). α-Glucosidase inhibition showed a rise and fall trend, with a maximum value of 47.84% at 7000 U/g. Considering both responses, 7000 U/g was chosen as the optimal enzyme dosage.

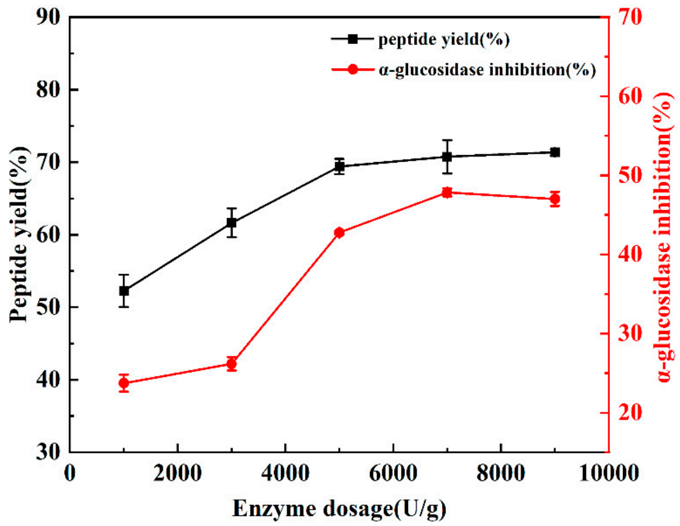

Fig. S2. Effect of enzyme dosage on peptide yield and α-glucosidase inhibition.

S1.3 Effect of pH

The influence of pH on peptide yield and α-glucosidase inhibition was examined at a substrate concentration of 6%, 50 °C, an enzyme dosage of 7000 U/g, and a hydrolysis time of 1 h (Fig. S3). Peptide yield decreased with increasing pH, while α-glucosidase inhibition first increased and then decreased. The highest inhibition (48.15%) was observed at pH 3. Thus, pH 3 was selected as the optimal hydrolysis pH.

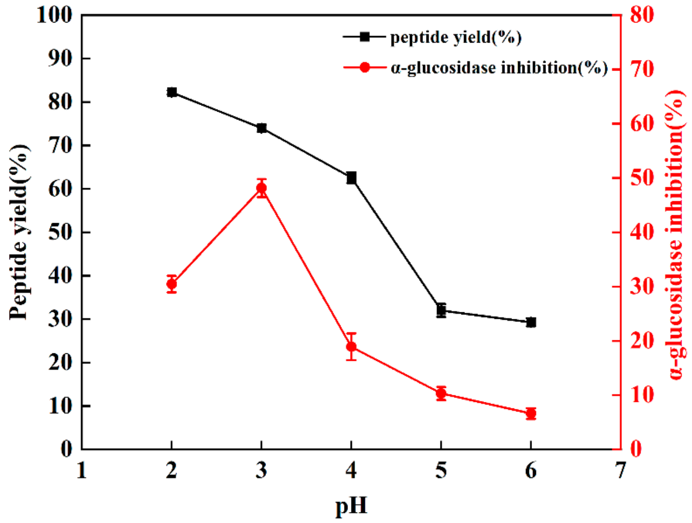

Fig. S3. Effect of pH on peptide yield and α-glucosidase inhibition.

S1.4 Effect of temperature

The effect of temperature on peptide yield and α-glucosidase inhibition was investigated at a substrate concentration of 6%, an enzyme dosage of 7000 U/g, pH 3, and a hydrolysis time of 1 h (Fig. S4). α-Glucosidase inhibition showed only minor changes between 30 and 70 °C, whereas peptide yield increased and then decreased, reaching a maximum of 74.59% at 50 °C. Therefore, 50 °C was selected as the optimal hydrolysis temperature.

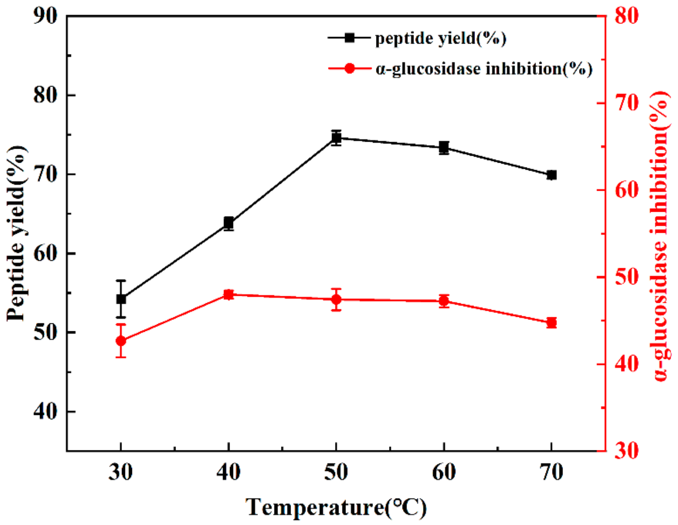

Fig. S4. Effect of temperature on peptide yield and α-glucosidase inhibition.

S1.5 Effect of hydrolysis time

The effect of hydrolysis time on peptide yield and α-glucosidase inhibition was studied at a substrate concentration of 6%, an enzyme dosage of 7000 U/g, 50 °C, and pH 3 (Fig. S5). In the range of 0.5-4 h, peptide yield increased slightly with time, while α-glucosidase inhibition first increased and then decreased. The maximum inhibition (53.14%) and a peptide yield of 77.54% were obtained at 3 h. Hence, 3 h was selected as the optimal hydrolysis time.

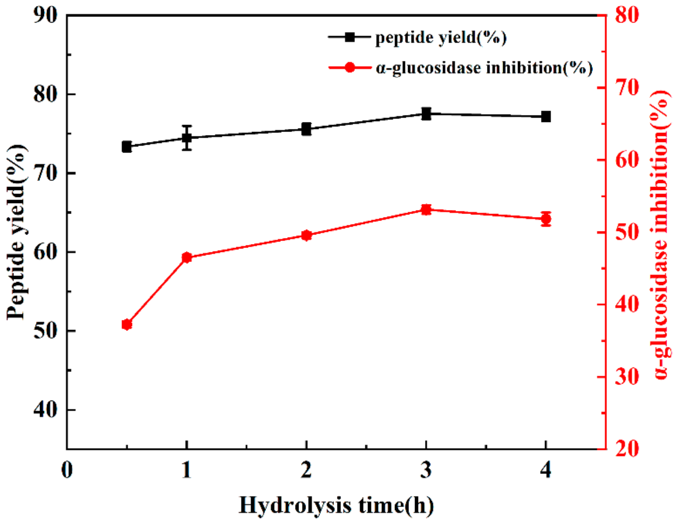

Fig. S5. Effect of hydrolysis time on peptide yield and α-glucosidase inhibition.

S2. Orthogonal optimization of hydrolysis conditions

An $L_9(3^4)$ orthogonal design was conducted with substrate concentration (A), enzyme dosage (B), pH (C), and hydrolysis time (D) as factors, and peptide yield and α-glucosidase inhibition as response variables (Table S1).

When peptide yield was used as the sole optimization index, range analysis indicated that the best combination was $A_3B_3C_1D_3$, corresponding to a substrate concentration of 6%, enzyme dosage of 7000 U/g, pH 2, and hydrolysis time of 3 h, giving a peptide yield of 84.87% and an α-glucosidase inhibition of 45.94%. When α-glucosidase inhibition was used as the sole index, the optimal combination was $A_1B_3C_2D_3$ (2% substrate, 7000 U/g enzyme, pH 3, 3 h), yielding the highest inhibition (55.88%) and a peptide yield of 75.51%.

Table S1. Orthogonal design and results for optimization of EMO peptide hydrolysis conditions.

No.		Factors				peptide yield (%)	α-glucosidase inhibition (%)
		substrate concentration (mg/mL)	enzyme dosage (U/g)	pH	hydrolysis time (h)		
1		1	1	1	1	59.49	6.89
2		1	2	2	2	73.12	42.15
3		1	3	3	3	60.89	53.99
4		2	1	2	3	71.52	40.50
5		2	2	3	1	47.39	19.65
6		2	3	1	2	83.87	39.03
7		3	1	3	2	56.82	9.00
8		3	2	1	3	83.92	34.89
9		3	3	2	1	73.10	42.61
peptide yield	K1	64.50	62.61	75.76	59.99		
	K2	67.59	68.14	72.58	71.27		
	K3	71.28	72.62	63.08	72.11		
	R1	6.78	10.01	12.68	12.12		
α-glucosidase inhibition	T1	34.34	18.80	26.94	23.05		
	T2	33.06	32.23	41.75	30.06		
	T3	28.83	45.21	27.54	43.13		
	R2	5.51	26.41	14.81	20.08		

Because optimization based on a single response (peptide yield vs. α-glucosidase inhibition) resulted in different preferred conditions, a comprehensive matrix analysis was carried out. Indicator matrices for α-glucosidase inhibition and peptide yield (M), together with the factor matrix (T) and level matrix (R), were constructed, and their product (W = MTR) was used as a weight matrix to evaluate the overall contribution of each factor-level combination. According to Eq. 1-8, the combination $A_2B_3C_2D_3$ showed the highest overall weight and was therefore selected as the optimal condition set, corresponding to a substrate concentration of 4%, an enzyme dosage of 7000 U/g, pH 3 and a hydrolysis time of 3 h. Under these conditions, α-glucosidase inhibition and peptide yield reached 56.30% and 86.54%, respectively, while reducing both raw material input and enzyme usage compared with the single-factor and single-index orthogonal optima, thus offering a more economical and practical process.

$$M_{\text{peptide yield}} = \begin{pmatrix} 64.50 & 0 & 0 & 0 \\ 67.59 & 0 & 0 & 0 \\ 71.28 & 0 & 0 & 0 \\ 0 & 62.61 & 0 & 0 \\ 0 & 68.14 & 0 & 0 \\ 0 & 72.62 & 0 & 0 \\ 0 & 0 & 75.76 & 0 \\ 0 & 0 & 72.58 & 0 \\ 0 & 0 & 63.08 & 0 \\ 0 & 0 & 0 & 59.99 \\ 0 & 0 & 0 & 71.27 \\ 0 & 0 & 0 & 72.11 \end{pmatrix} \tag{S1}$$

$$M_{\alpha\text{-glucosidase inhibition}} = \begin{pmatrix} 34.09 & 0 & 0 & 0 \\ 33.06 & 0 & 0 & 0 \\ 28.83 & 0 & 0 & 0 \\ 0 & 18.80 & 0 & 0 \\ 0 & 32.23 & 0 & 0 \\ 0 & 45.21 & 0 & 0 \\ 0 & 0 & 26.94 & 0 \\ 0 & 0 & 41.75 & 0 \\ 0 & 0 & 27.54 & 0 \\ 0 & 0 & 0 & 23.05 \\ 0 & 0 & 0 & 30.06 \\ 0 & 0 & 0 & 43.13 \end{pmatrix} \tag{S2}$$

$$T_{\text{peptide yield}} = \begin{pmatrix} 0.0049 & 0 & 0 & 0 \\ 0 & 0.0049 & 0 & 0 \\ 0 & 0 & 0.0047 & 0 \\ 0 & 0 & 0 & 0.0049 \end{pmatrix} \tag{S3}$$

$$T_{\alpha\text{-glucosidase inhibition}} = \begin{pmatrix} 0.0105 & 0 & 0 & 0 \\ 0 & 0.0104 & 0 & 0 \\ 0 & 0 & 0.0104 & 0 \\ 0 & 0 & 0 & 0.0104 \end{pmatrix} \tag{S4}$$

$$R_{\text{peptide yield}} = \begin{pmatrix} 0.1630 \\ 0.2407 \\ 0.3049 \\ 0.2914 \end{pmatrix} \tag{S5}$$

$$R_{\alpha\text{-glucosidase inhibition}} = \begin{pmatrix} 0.0825 \\ 0.3953 \\ 0.2217 \\ 0.3006 \end{pmatrix} \tag{S6}$$

$$W_{\text{peptide yield}}=\begin{pmatrix}0.0515\\0.0540\\0.0569\\0.0739\\0.0804\\0.0857\\0.1086\\0.1040\\0.0904\\0.0857\\0.1018\\0.1029\end{pmatrix}\quad W_{\alpha\text{-glucosidase inhibition}}=\begin{pmatrix}0.0295\\0.0286\\0.0250\\0.0773\\0.1325\\0.1859\\0.0621\\0.0963\\0.0635\\0.0793\\0.0940\\0.1348\end{pmatrix} \tag{S7}$$

$$W=\frac{W_{\text{peptide yield}}+W_{\alpha-\text{glucosidase inhibition}}}{2}=\begin{pmatrix}0.0405\\0.0413\\0.0410\\0.0756\\0.1065\\0.1358\\0.0854\\0.1002\\0.0770\\0.0825\\0.0979\\0.1189\end{pmatrix} \tag{S8}$$
